# Immune Phenotyping Using Neutrophil-to-Lymphocyte Ratio and Tumor-Infiltrating Lymphocytes Predicts Recurrence in Resected Melanoma

**DOI:** 10.3390/diagnostics16101495

**Published:** 2026-05-14

**Authors:** Omer Ekin, Oktay Halit Aktepe

**Affiliations:** 1 Private Practice, Ankara 06800, Türkiye; 2 Department of Medical Oncology, Dokuz Eylul University, Izmir 35330, Türkiye

**Keywords:** cutaneous melanoma, neutrophil-to-lymphocyte ratio, prognosis, recurrence-free survival, tumor-infiltrating lymphocyte

## Abstract

**Background and Objectives:** Tumor-infiltrating lymphocytes (TIL) and the neutrophil-to-lymphocyte ratio (NLR) are each associated with prognosis in melanoma, yet their combined prognostic value remains insufficiently defined. We aimed to assess whether integrating NLR and TILs into a combined immune phenotype improves prediction of recurrence-free survival (RFS) in patients with resected cutaneous melanoma. **Materials and Methods:** A total of 203 patients were included. Receiver operating characteristic analysis identified an NLR cut-off of 2.75 for RFS, defining low (<2.75) and high (≥2.75) groups. TIL status was dichotomized as present or absent. According to the combined NLR–TIL profile, patients were initially categorized into three immune phenotypes: favorable (low NLR and TIL-positive), intermediate (low NLR and TIL-negative or high NLR and TIL-positive), and unfavorable (high NLR and TIL-negative). For the dichotomized analysis, the intermediate and unfavorable phenotypes were combined and compared with the favorable phenotype. Associations of clinicopathological factors with RFS were evaluated using Kaplan–Meier curves and Cox regression models. **Results:** The median follow-up was 56 months. In the univariate analysis, stage III disease, greater Breslow thickness, increased mitotic rate, and absence of adjuvant therapy were associated with worse RFS. In addition, patients with an unfavorable immune phenotype had a markedly increased risk of recurrence compared with those in the favorable group (HR 2.86, 95% CI 1.43–5.71; *p* = 0.004). In multivariate Cox regression analysis, both the unfavorable immune phenotype and stage III disease independently predicted RFS (HR 2.25, 95% CI 1.11–4.54; *p* = 0.024 and HR 2.13, 95% CI 1.03–4.43; *p* = 0.041, respectively). **Conclusions:** Combined assessment of systemic inflammation and tumor-local immune response using NLR and TILs may provide meaningful prognostic stratification in resected cutaneous melanoma.

## 1. Introduction

Melanoma is an aggressive form of skin cancer with rising incidence worldwide and accounts for the majority of skin cancer-related deaths [1]. Clinical outcomes are strongly stage-dependent, with morbidity and mortality increasing markedly in advanced disease [2]. This highlights the need for improved post-surgical risk stratification to identify patients at high risk of recurrence.

Host immune response has emerged as a critical factor influencing melanoma outcomes. Tumor-infiltrating lymphocytes (TILs), the immune cells present within the tumor microenvironment, have long been recognized as an indicator of the host’s anti-tumor immune activity [3]. For example, a large multi-institutional analysis of 4957 melanoma patients found TIL presence to be strongly prognostic of melanoma-specific survival, overall survival (OS), and recurrence-free survival (RFS) [4], underscoring their role as indicators of effective local anti-tumor immunity. However, despite this positive association, some studies have noted that TIL status is not always a stand-alone prognostic factor on multivariate analysis when controlling for other variables like tumor thickness or ulceration [5,6,7]. Although TILs are routinely documented in pathology reports, TILs have not been incorporated into the American Joint Committee on Cancer (AJCC) staging system despite routine reporting in melanoma pathology.

Complementing local immune factors, systemic inflammation markers—particularly the neutrophil-to-lymphocyte ratio (NLR)—have emerged as easily obtainable, cost-effective prognostic indicators in melanoma and other cancers. An elevated NLR reflects a protumor inflammatory state characterized by enhanced tumor-promoting neutrophil activity and impaired lymphocyte-mediated immunity, and has been linked to poorer outcomes in various cancers including melanoma [8,9,10,11,12,13]. In a 2018 meta-analysis pooling 12 melanoma studies and 3207 patients, high NLR was associated with significantly shorter OS and progression-free survival compared with low NLR [14].

Collectively, TILs and NLR provide complementary prognostic information, as TILs represent the local tumor immune microenvironment whereas NLR reflects systemic inflammation and immune balance [15,16]. Importantly, local and systemic immune signals are not necessarily concordant; patients may exhibit prominent intratumoral lymphocytic infiltration despite an unfavorable systemic inflammatory profile, or conversely show a relatively favorable systemic index in the absence of meaningful local immune engagement. Accordingly, integrating NLR with TIL status may offer a more comprehensive characterization of the host–tumor immune context and improve postoperative recurrence-risk stratification. Therefore, in patients with resected cutaneous melanoma, we evaluated the prognostic relevance of a combined NLR–TIL immune phenotype as a practical approach to postoperative recurrence-risk assessment.

## 2. Materials and Methods

### 2.1. Patients and Study Design

**Figure 1:**
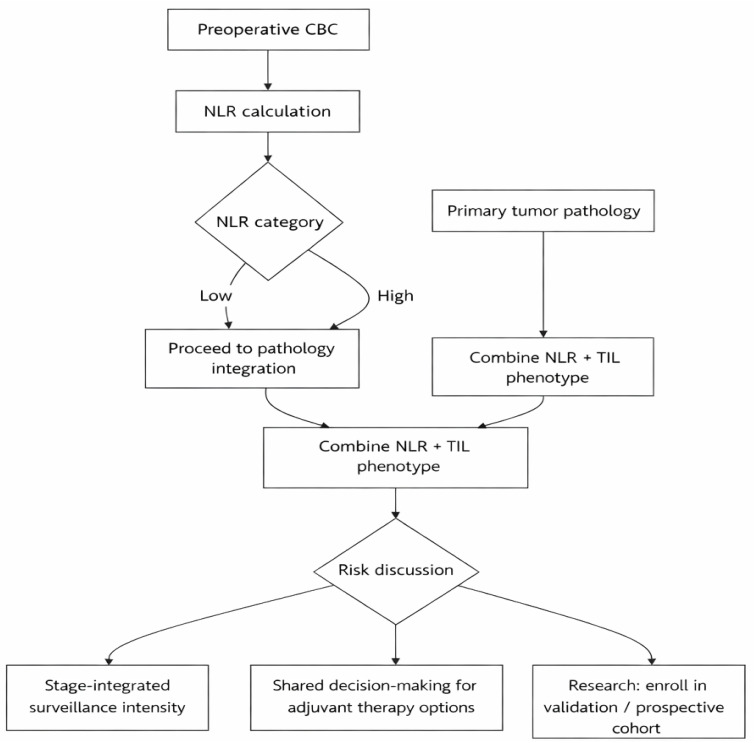
Flow diagram of patient selection and study cohort formation.

The overall study design is summarized in Figure 1. This retrospective cohort study included patients with histologically confirmed cutaneous melanoma who were managed at Dokuz Eylul University (Izmir, Türkiye) and the Omer Ekin Private Clinic (Ankara, Türkiye) between January 2015 and January 2025. Patients referred for follow-up after receiving treatment elsewhere were also included. Demographic, clinicopathological, laboratory, and follow-up data were retrieved from electronic medical records, including age, sex, primary tumor location, Breslow thickness, Clark level, mitotic rate, ulceration status, tumor stage (I–II vs. III), and receipt of adjuvant therapy. Tumor staging was performed according to the American Joint Committee on Cancer 8th edition staging system [2]. Eligible patients had histologically confirmed cutaneous melanoma and had a preoperative peripheral blood count available within 14 days prior to surgery. Patients were excluded if they had evidence of distant metastatic disease at diagnosis, received neoadjuvant systemic therapy, had concomitant conditions or treatments likely to substantially affect peripheral blood counts at the time of baseline sampling (e.g., active infection/inflammatory disease or systemic corticosteroid/immunosuppressive therapy), or had missing key data required for immune phenotyping or survival analyses.

For each patient, we calculated the NLR from the preoperative complete blood count differential, defined as the absolute neutrophil count divided by the absolute lymphocyte count. We then determined the optimal cut-off value for high versus low NLR using receiver operating characteristic (ROC) curve analysis. TIL information was obtained from routine histopathology reports on the resected primary tumors. Clark’s TIL classification, which remains widely used, categorizes TILs as present or absent and further distinguishes their extent as brisk or non-brisk based on microscopic density [4]. TIL status was simplified into a binary variable, with tumors classified as TIL-positive when any infiltrating lymphocytes were reported and as TIL-negative when no TILs were identified. For the initial analysis, patients were stratified into three prognostic immune categories: favorable (low NLR/TIL-positive), intermediate (low NLR/TIL-negative or high NLR/TIL-positive), and unfavorable (high NLR/TIL-negative). In subsequent dichotomized analyses, patients with intermediate or unfavorable immune phenotypes were combined into a single group and compared with patients with a favorable immune phenotype.

### 2.2. Statistical Analysis

Continuous variables were summarized as median and interquartile range (IQR) and compared using the Mann–Whitney U test. Categorical variables were presented as frequencies and percentages and compared using the chi-square test or Fisher’s exact test, as appropriate. RFS was estimated using the Kaplan–Meier method, and survival curves were compared with the log-rank test. The primary endpoint was RFS, defined as the interval from surgery to the first documented recurrence or death from any cause, with censoring at last follow-up for event-free patients. Univariable Cox proportional hazards regression analyses were performed to evaluate associations between clinicopathological variables and RFS. Variables with clinical relevance and/or statistical significance in univariable analyses were entered into a multivariable Cox regression model. To avoid model overfitting, the number of covariates was limited in accordance with the number of observed events. Hazard ratios (HRs) were reported with 95% confidence intervals (CIs). Survival curves were generated using R software (version 4.5.2; R Foundation for Statistical Computing, Vienna, Austria), and all other statistical analyses were performed using SPSS software (version 27.0; IBM Corp., Armonk, NY, USA). A two-sided *p* value < 0.05 was considered statistically significant.

## 3. Results

### 3.1. Patient Characteristics

**Figure 2:**
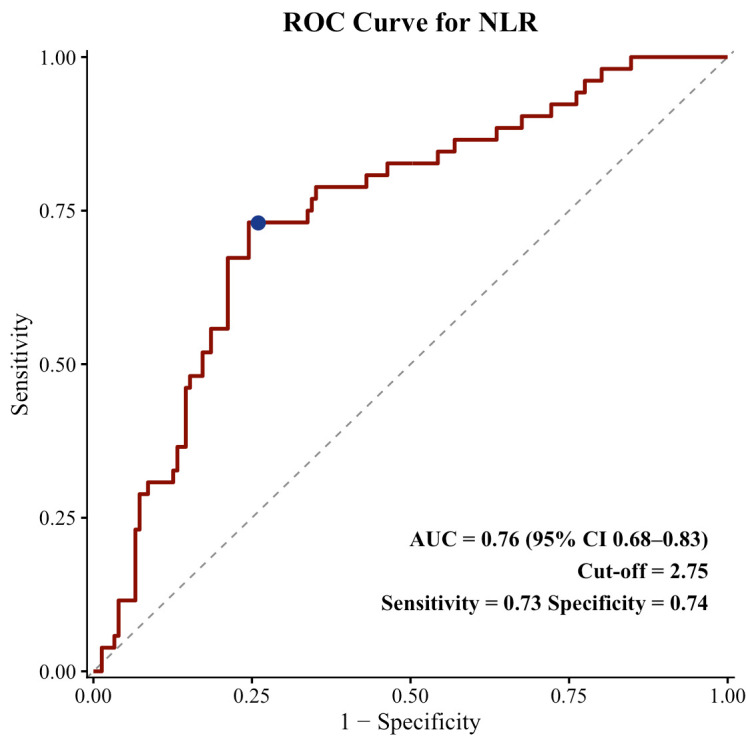
ROC curve of NLR for RFS. The dashed line represents the reference line, and the blue dot indicates the selected optimal NLR cut-off point of 2.75.

Overall, a total of 203 patients with resected melanoma were included in the study. As shown in Figure 2, ROC analysis identified an NLR cut-off of 2.75 for RFS [area under the curve (AUC): 0.76, sensitivity 0.73, specificity 0.74], which was used to dichotomize patients into low NLR (<2.75) and high NLR (≥2.75) groups. As described in Section 2, patients were classified into favorable and unfavorable groups according to the combined NLR–TIL profile. Baseline characteristics of the study population stratified by prognostic subgroups are presented in Table 1. The median age of the entire cohort was 59 years (IQR: 48–68), and 55.7% of patients were male. The median Breslow thickness was 2.5 mm (IQR: 1.0–5.2), and 46.8% of tumors were ulcerated. Most patients had Clark level IV–V disease (62.6%), and the median mitotic rate was 5/mm^2^ (IQR: 2–12). Regarding disease stage, 56.1% of patients had stage I–II disease, while 43.9% were stage III. Primary tumors were most commonly located in the extremities (42.9%), followed by the trunk (32.0%) and head and neck region (25.1%). Adjuvant therapy was administered in 29.1% of patients. Among the 59 patients who received adjuvant therapy, 37 (62.7%) were treated with immunotherapy (nivolumab, ipilimumab, or pembrolizumab), 13 (22.0%) received interferon, and 9 (15.3%) received dabrafenib plus trametinib. In the favorable group, adjuvant treatment consisted of immunotherapy in 19 patients (82.6%), interferon in 1 patient (4.3%), and dabrafenib plus trametinib in 3 patients (13.0%). In the unfavorable group, the corresponding numbers were 18 (50.0%), 12 (33.3%), and 6 (16.7%), respectively.

When stratified according to immune phenotype, 84 patients (41.4%) were classified as the favorable group, and 119 patients (58.6%) as the unfavorable group. Baseline demographic characteristics, including age and sex distribution, were comparable between the two groups. Similarly, no statistically significant differences were observed with respect to Clark level, tumor stage, primary tumor location, or receipt of adjuvant therapy (all *p* > 0.05). Tumors in the unfavorable group tended to exhibit a greater Breslow thickness (median 2.8 vs. 1.8 mm, *p* = 0.062) and a higher mitotic rate (median 6/mm^2^ vs. 4/mm^2^, *p* = 0.077), although these differences did not reach statistical significance. In contrast, ulceration was significantly more frequent in the unfavorable group compared with the favorable group (52.9% vs. 38.1%, *p* = 0.037).

### 3.2. The Predictors of RFS

**Figure 3:**
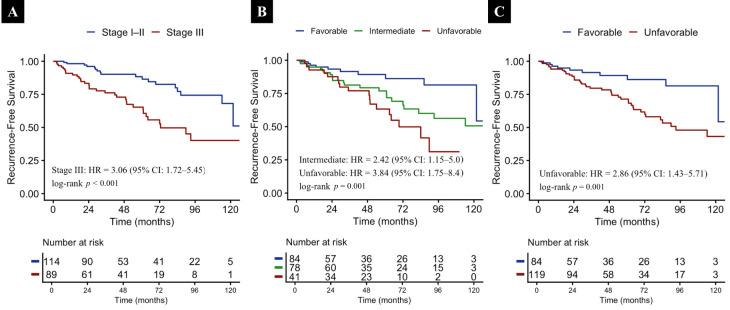
Kaplan–Meier curves for recurrence-free survival according to disease stage (**A**), the three-group combined NLR–TIL immune phenotype (**B**), and the dichotomized combined NLR–TIL immune phenotype (**C**). Survival differences were assessed using the log-rank test, and HRs with 95% CIs were derived from Cox proportional hazards regression models.

During a median follow-up of 56 months, 52 recurrences were observed. The median RFS for the entire cohort was 121.7 months (95% CI 106.9–136.5). Patients with stage III melanoma experienced significantly shorter RFS than those with stage I–II disease (Figure 3A, *p* < 0.001). Median RFS was reached only in stage III patients at 72.6 months, whereas stage I–II patients did not reach median RFS during follow-up. When RFS was analyzed according to the combined NLR–TIL immune phenotype, significant differences were observed across both the three-category and dichotomized subgroup classifications. In the three-category analysis, RFS differed significantly among the favorable, intermediate, and unfavorable groups (Figure 3B, *p* = 0.001). Median RFS was not reached in the favorable group, whereas it was 207.4 months in the intermediate group and 69.4 months (95% CI 46.7–92.0) in the unfavorable group. Consistent with this finding, the dichotomized analysis also showed a significant difference between the favorable and unfavorable groups (Figure 3C, *p* = 0.001). Median RFS was not reached in the favorable group, whereas it was 93.1 months (95% CI 60.7–125.4) in the unfavorable group.

Univariate Cox regression analyses were performed to evaluate the association between clinicopathological variables and RFS (Table 2). In univariate analyses, Breslow thickness was significantly associated with an increased risk of recurrence (HR 1.06, 95% CI 1.02–1.11; *p* = 0.004). Similarly, a higher mitotic rate was associated with worse RFS (HR 1.01, 95% CI 1.00–1.02; *p* = 0.044). Patients with stage III disease had a markedly higher risk of recurrence compared with those with stage I–II disease (HR 3.06, 95% CI 1.72–5.45; *p* < 0.001). Absence of adjuvant therapy was also associated with poorer RFS in univariate analysis (HR 2.46, 95% CI 1.40–4.33; *p* = 0.002). In univariate analysis, both the three-category and dichotomized combined NLR–TIL immune phenotypes were significantly associated with RFS. For the three-category classification, compared with the favorable group, the intermediate group was associated with an increased risk of recurrence (HR 2.42, 95% CI 1.15–5.00), and the unfavorable group was also associated with an increased risk of recurrence (HR 3.84, 95% CI 1.75–8.40) (overall *p* = 0.003). Similarly, in the dichotomized analysis, patients classified in the unfavorable group had significantly shorter RFS than those in the favorable group (HR 2.86, 95% CI 1.43–5.71; *p* = 0.004).

**Figure 4:**
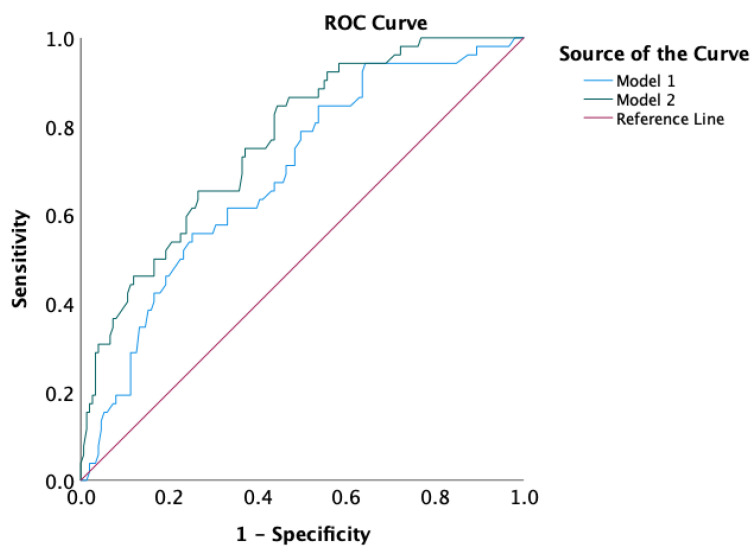
ROC curves comparing Model 1, including stage, Breslow thickness, and Clark level, with Model 2, which also included the combined NLR–TIL phenotype. The AUC was 0.690 (95% CI: 0.609–0.770) for Model 1 and 0.767 (95% CI: 0.697–0.838) for Model 2.

Variables with clinical relevance and/or statistical significance in univariate analyses were subsequently entered into a multivariate Cox regression model, in which the dichotomized combined NLR–TIL immune phenotype was used. As shown in Table 2, the unfavorable immune phenotype remained an independent predictor of RFS in the multivariate analysis (HR 2.25, 95% CI 1.11–4.54; *p* = 0.024). Additionally, tumor stage (III vs. I–II) was an independent prognostic variable in predicting RFS (HR 2.13, 95% CI 1.03–4.43; *p* = 0.041). After multivariate adjustment, Breslow thickness, mitotic rate, and adjuvant therapy were not independently associated with RFS. To evaluate and compare model performance, ROC analysis was performed (Figure 4). The baseline model incorporating stage, Breslow thickness, and Clark level achieved an AUC of 0.690 (95% CI 0.609–0.770), whereas the extended model further including the combined NLR–TIL phenotype achieved an AUC of 0.767 (95% CI 0.697–0.838), indicating improved discriminative ability. Both models performed significantly better than chance (each *p* < 0.001).

## 4. Discussion

This study evaluated the prognostic value of integrating NLR and TILs in resected cutaneous melanoma and demonstrates that this combined immune phenotype provides clinically meaningful stratification of recurrence risk. Notably, patients exhibiting low NLR with TILs achieved significantly better RFS compared with patients lacking tumor-infiltrating lymphocytes and showing elevated NLR. After adjustment for relevant covariates, the combined NLR and TILs phenotype remained independently associated with RFS in multivariable analysis.

These results are clinically significant because they underscore the complementary roles of systemic inflammation and tumor-localized immunity in melanoma progression. Prior studies have individually shown the importance of each of these factors: elevated NLR has been repeatedly linked to worse outcomes [17,18,19], and the presence of TILs generally portends a more favorable prognosis [20,21]. However, our study is one of the first melanoma studies to evaluate both simultaneously. We show that considering them in combination can refine risk assessment. For example, we found that among patients with elevated NLR, those with TILs experienced more favorable outcomes than those lacking TILs, suggesting that a robust local immune response may mitigate the adverse effects of systemic inflammation. Conversely, patients with low NLR were not uniformly low-risk, as those whose tumors lacked TILs experienced higher recurrence rates than low-NLR patients with TIL-positive tumors, indicating that the absence of a local immune response can offset the prognostic advantage of a favorable systemic profile. Thus, each marker alone is insufficient, and their combined interaction provides more meaningful prognostic insight. This is consistent with evidence from other malignancies showing that composite immune scores provide superior prognostic performance compared with single markers [22,23]. For example, in colon cancer, the combined assessment of systemic inflammation and tumor immune infiltration demonstrated superior prognostic value compared with either parameter alone, with patients characterized by low NLR and high CD8^+^ TILs showing the most favorable survival outcomes [22]. Likewise, in locally advanced nasopharyngeal carcinoma, a combined index of intratumoral TIL density and peripheral lymphocyte count stratified patients into distinct risk groups more effectively than either factor alone [23].

From a mechanistic perspective, the prognostic discrimination achieved by the NLR–TIL phenotype aligns with an immunobiologic model where systemic myeloid skewing and local T-cell competence are coupled, but not interchangeable [24,25]. High NLR, capturing neutrophilia with relative lymphopenia, may indicate predominance of innate myeloid activity that could support tumor progression beyond simply mirroring systemic inflammation [25,26]. Neutrophils can contribute to tumor progression by promoting immunosuppression, angiogenesis, and metastatic spread [26]. Chronic cancer-related inflammation can promote expansion of myeloid-derived suppressor cells, including granulocytic/neutrophil-like subsets, which suppress T-cell responses through arginase-1-mediated metabolic depletion, inducible nitric oxide synthase activity, and reactive oxygen/nitrogen species, leading to impaired effector lymphocyte function [24,27,28]. In this systemic context, TIL status indicates the level of local adaptive immune involvement [29]. Therefore, a high NLR + TIL-negative pattern may indicate both systemic myeloid skewing and weak local T-cell activity, which could lessen immune surveillance of minimal residual disease.

Recurrence following resection may also be mechanistically linked to neutrophil activation through the generation of neutrophil extracellular traps (NET), extracellular DNA–protein webs formed in inflammatory settings [30,31]. Experimental studies suggest that NETs can trap circulating tumor cells and promote micrometastatic seeding and metastatic growth in vivo; these effects lessen when NETs are inhibited [30,31]. These data do not establish NETs as the dominant pathway for recurrence in resected melanoma. However, they support the biological plausibility that a neutrophil-high systemic state (reflected by high NLR) may favor tumor dissemination and be associated with broader immune suppression that limits effective T-cell control. Taken together, this framework helps explain why high NLR is most unfavorable when TILs are absent. In that setting, both systemic and local immunity shift toward immune escape rather than immune control.

Several limitations should be considered when interpreting our findings. First, the retrospective and two-center design introduces the possibility of selection bias and residual confounding, despite multivariable adjustment for established clinicopathologic factors. The relatively modest cohort size and limited number of recurrence events may have contributed to model instability and limited generalizability. In addition, incomplete availability of key molecular and treatment-related variables, including BRAF mutation status, may have further affected interpretation of the findings. Second, TIL status was derived from routine pathology reports and dichotomized as present or absent for analytic feasibility. While pragmatic, this approach may not fully capture the biological complexity of the tumor immune microenvironment. Interobserver variability and the lack of standardized quantitative or immunophenotypic characterization may have led to misclassification. More refined tissue-level immunophenotyping—distinguishing CD8^+^ effector infiltration, exhausted T-cell phenotypes, regulatory T-cell enrichment, and myeloid signatures—could clarify the immune programs underlying TIL-negative biology and determine whether systemic myeloid skewing parallels intratumoral suppressive states. Such efforts would move this phenotype from a pragmatic surrogate toward a more mechanistically anchored model. Third, NLR was derived from a single preoperative blood sample and dichotomized using a cohort-specific ROC-based cut-off of 2.75, which may not fully reflect the continuous nature of the marker and may capture transient inflammatory fluctuations rather than a stable host immune phenotype. In addition, the use of a relatively low cohort-derived NLR threshold together with a simplified binary TIL classification may have limited biological resolution and reduced the broader clinical utility and generalizability of the proposed immune phenotype. Fourth, although common staging criteria and uniform study definitions were applied, pathology reporting and treatment decisions were not prospectively standardized across centers, and residual inter-center variability may have been present. This is particularly relevant for TIL assessment, which was based on routine pathology reports and simplified into a binary present/absent variable, potentially limiting biological resolution and introducing some degree of misclassification. Finally, some patients were referred from outside institutions after completion of their initial pathological assessment and were subsequently managed at the participating centers for follow-up and treatment planning. This may have introduced referral-related selection bias and additional heterogeneity arising from differences in pre-referral diagnostic evaluation and clinical management.

## 5. Conclusions

In resected cutaneous melanoma, the combined NLR–TIL phenotype may offer complementary prognostic information beyond standard clinicopathologic factors. Nevertheless, these findings should be considered exploratory, as the absence of an independent validation cohort limits broader interpretation. External validation in larger, preferably multicenter, cohorts will be required before this approach can be considered for routine clinical use.

## Figures and Tables

**Table 1:** Baseline patient characteristics stratified according to prognostic subgroups.

Characteristics	All Patients (*n* = 203)	Favorable (*n* = 84, 41.4%)	Unfavorable (*n* = 119, 58.6%)	*p* Value
Age, years (IQR)	59 (48–68)	62 (49–69)	56 (46–67)	0.201
Sex				0.352
Female	90 (44.3%)	34 (40.5%)	56 (47.1%)	
Male	113 (55.7%)	50 (59.5%)	63 (52.9%)	
Breslow thickness, (IQR)	2.5 (1.0–5.2)	1.8 (0.8–5.0)	2.8 (1.5–5.5)	0.062
Clark				0.871
II–III	76 (37.4%)	32 (38.1%)	44 (37%)	
IV–V	127 (62.6%)	52 (61.9%)	75 (63%)	
Mitotic rate, (IQR)	5 (2–12)	4 (1–11)	6 (2–12)	0.077
Ulceration				0.037
No	108 (53.2%)	52 (61.9%)	56 (47.1%)	
Yes	95 (46.8%)	32 (38.1%)	63 (52.9%)	
Tumor stage				0.600
I–II	114 (56.1%)	49 (58.3%)	65 (54.6%)	
III	89 (43.9%)	35 (41.7%)	54 (45.4%)	
Tumor location				0.514
Trunk	65 (32%)	29 (34.5%)	36 (30.3%)	
Extremity	87 (42.9%)	32 (38.1%)	55 (46.2%)	
Head and neck	51 (25.1%)	23 (27.4%)	28 (23.5%)	
Adjuvant therapy				0.657
No	144 (70.9%)	61 (72.6%)	83 (69.7%)	
Yes	59 (29.1%)	23 (27.4%)	36 (30.3%)	

Abbreviations: IQR: Interquartile Range.

**Table 2:** Univariate and multivariate Cox proportional hazards analyses of candidate prognostic variables for RFS.

	Univariate		Multivariate	
Variable	HR (95% CI)	*p* Value	HR (95% CI)	*p* Value
Age, years	0.99 (0.97–1.01)	0.474	NA	NA
Gender (male vs. female)	0.94 (0.54–1.65)	0.850	NA	NA
Breslow	1.06 (1.02–1.11)	0.004	1.03 (0.97–1.08)	0.308
Mitotic rate	1.01 (1–1.02)	0.044	1.0 (0.99–1.01)	0.526
Stage (III vs. I–II)	3.06 (1.72–5.45)	<0.001	2.13 (1.03–4.43)	0.041
Adjuvant therapy (no vs. yes)	2.46 (1.40–4.33)	0.002	1.43 (0.74–2.75)	0.280
Unfavorable vs. favorable	2.86 (1.43–5.71)	0.004	2.25 (1.11–4.54)	0.024

Abbreviations: HR: hazard ratio; CI: confidence interval; NA: not applicable.

## Data Availability

The data are not publicly available due to privacy and ethical restrictions related to patient confidentiality but are available from the corresponding author upon reasonable request.

## Abbreviations

The following abbreviations are used in this manuscript:

AUC	Area under the curve
CI	Confidence Interval
HR	Hazard Ratio
IQR	Interquartile Range
NET	Neutrophil Extracellular Trap
NLR	Neutrophil-to-Lymphocyte Ratio
OS	Overall Survival
RFS	Recurrence-Free Survival
ROC	Receiver Operating Characteristic
TIL(s)	Tumor-Infiltrating Lymphocyte(s)
